# Mechanochemically Activated Ternary Modifier Based on Technogenic Mineral Waste for High-Performance Concrete

**DOI:** 10.3390/ma19194164

**Published:** 2026-09-29

**Authors:** Ruslan E. Nurlybayev, Axaya S. Yestemessova, Zaure N. Altayeva, Yelzhan S. Orynbekov, Aktota A. Murzagulova, Maxat Zh. Bulenbayev, Bakytkul U. Yerkebayeva, Indira B. Tashmukhanbetova

**Affiliations:** 1 K.I. Satbayev Kazakh National Research Technical University, Almaty 050000, Kazakhstan; nurlybayev.savenergy@gmail.com (R.E.N.); zaltaeva@mail.ru (Z.N.A.); aktoty_sun@mail.ru (A.A.M.); mbulenbaev@mail.ru (M.Z.B.); 2 International Educational Corporation LLP, Almaty 050043, Kazakhstan; 3 Limited Liability Partnership “Center for Confirmation of Product Conformity”, Almaty 050062, Kazakhstan; bakytkul-e@yandex.ru

**Keywords:** modified concrete, modifying mineral additive, waste from technogenic deposits, chemical additive, frost resistance, water resistance, mechanochemical activation

## Abstract

**Highlights:**

- A ternary modifier was developed from mineral processing tailings, shungite waste, and silica fume;
- Steel cylpebs improved grinding efficiency by 1.9% and reduced activation time to 30 min;
- The selected 59:40:1 modifier absorbed 59.2% CaO; an 8% dosage increased strength by 16%;
- Modified concrete achieved a freeze–thaw resistance grade of F400 and a water impermeability grade of W14;
- Electron microscopy, XRD, and DTA revealed the formation of calcium silicate hydrates with higher basicity and stable hydrate phases due to the pozzolanic reaction and the presence of nanosized particles.

**Abstract:**

This article presents the results of a research study on the development and evaluation of a mineral modifying additive for concrete produced from mineral processing tailings, shale waste from shungite production, and silica fume. Compositions of both the modifying additive and concretes based on it were developed. The properties of the mineral modifying additive were studied; mechanochemical activation was applied to increase and modify its activity, and its pozzolanic activity was determined. Depending on the component ratio, CaO absorption ranged from 7.9% to 94%. In the technology of high-strength and functional concretes, high-carbon slag with a fraction of 10–20 mm was used as a partial replacement for natural coarse aggregate. The introduction of 3–15% mineral modifying additive into B40 concrete increased the 28-day compressive strength by 0.19–16%. The greatest increase was observed for concrete containing 8% mineral modifying additive by cement mass. Because compressive strength is not directly correlated with freeze–thaw resistance and water impermeability, capillary suction tests were performed. This non-destructive durability indicator makes it possible to assess concrete permeability, characterize the pore structure, indirectly evaluate freeze–thaw resistance through water-absorption behavior, and estimate the potential resistance of concrete to repeated freezing and thawing. After 24 h, the maximum increase in capillary suction ranged from 4.13% to 5.17%. Direct freeze–thaw testing confirmed a freeze–thaw resistance grade of F400. Three main criteria were used to assign the freeze–thaw resistance grade: average mass loss not exceeding 2%; compliance with the compressive strength ratio XminII >= 0.9 XminI; and the absence of cracks, spalling, and edge-scaling on the specimens.

## 1. Introduction

Modern construction places high demands on concrete quality, which increases the demand for modified concretes. This trend is associated with the need to improve concrete strength and durability, develop new technical and structural solutions, and increase the reliability and environmental performance of buildings. Complex modifiers are an effective tool for regulating and controlling the properties of both precast and cast-in-place concrete [1,2,3,4,5,6,7,8].

The development of technologies for producing complex multicomponent modifiers for cast-in-place concrete is an important scientific and practical task. Its solution, through the use of chemical raw materials and mineral wastes, can support the development and practical utilization of technogenic mineral resources.

The rational use of mining and mineral processing wastes as technogenic resources is not a new topic. Problems associated with the rational use of mineral resources, including wastes from extraction and processing, have long been studied. The specific feature of the present approach is that mining and processing wastes are considered in terms of their dual nature: as secondary mineral resources and as sources of negative environmental impact in the context of greening industrial production. The need to address the rational use of secondary mineral resources is explained by several factors. First, environmental regulation is shifting toward systems based on the best available technologies. This approach changes the institutional framework for managing production and consumer wastes, including the waste management practices of mining enterprises. Second, geological, ore-quality, and other sector-specific problems in mining are increasing. The deterioration of natural mineral raw material quality, more difficult extraction conditions, and the depletion of developed deposits mean that the quality of natural mineral resources is becoming comparable with that of accumulated anthropogenic raw materials. As a result, the proportion of waste in extracted raw materials increases and creates conditions for an inevitable transition to a new level of resource use. Third, the problem of integrated ore utilization remains unresolved. Most ores have a multicomponent composition, whereas deposits are commonly developed as if they contained a single target resource. Users extract the main mineral resource, while associated minerals and valuable components enter mining and primary processing wastes. Consequently, uncontrolled waste accumulation continues, with a tendency toward increasing accumulation rates despite efforts to improve the utilization and recycling of mining and associated processing wastes [5,6,7,8,9,10,11,12].

According to the Bureau of National Statistics of the Agency for Strategic Planning and Reforms of the Republic of Kazakhstan, more than 22 billion tons of waste have accumulated in the country, including more than 16 billion tons of technogenic mineral formations. On average, approximately 700 million tons of industrial waste are generated annually. Statistical data also indicate that the waste utilization rate remains within 3–7%.

Wastes from mining and industrial enterprises are an important source of raw materials for the construction industry. According to available data, approximately 20 billion tons of mining waste may be suitable for the production of construction materials, including at least 30% of waste rock and enriched sludges, as well as nearly all metallurgical and fuel slags. A large proportion of waste rock from iron-ore deposits is suitable for producing crushed stone, cement, and ceramic materials.

In terms of technological parameters and physical–mechanical properties, mining and industrial wastes are not inferior to natural construction raw materials, while the volumes of technogenic raw material wastes greatly exceed the needs of the construction sector. However, their active involvement in the economic cycle is limited by low demand in the areas where technogenic deposits are located and by other factors, particularly high transport costs for supplying remote regions.

The total area of land occupied by waste exceeds 1000 thousand km^2^, of which more than 0.6 thousand km^2^ is occupied by tailings storage facilities and sludge ponds, and more than 1 thousand km^2^ by waste rock dumps, ash dumps, and slag dumps. Each year, the area of disturbed land increases by at least 85–90 km^2^. Long-term open-air storage under atmospheric exposure releases useful components into the environment, where they become pollutants, thereby increasing the risk of surface- and groundwater contamination, vegetation cover degradation, and landscape structure changes.

The generation and accumulation of mining waste are not only problems of individual mining enterprises; their national scale is demonstrated by official statistics (Figure 1). Industrial wastes account for more than 96% of all production and consumption wastes recorded in national statistics. More than 90% of the annual total volume of production and consumption wastes of all hazard classes is generated during natural resource extraction, with more than 60% related to the extraction of energy resources and fuels. Wastes from the processing industry account for approximately 5% of the total waste generated, more than half of which is associated with metallurgy and the manufacture of finished metal products. Among industrial sectors, the main generators and accumulators of waste are coal mining and ferrous and non-ferrous metallurgy.

Statistics for Kazakhstan indicate a sharp decrease in the share of industrial waste processing and utilization. According to the 2023 results of the national project Green Kazakhstan, this indicator was 30.4%, which is 10% lower than in 2022. At the same time, the Ministry of Ecology and Natural Resources of the Republic of Kazakhstan planned to increase the share of the processing of industrial tailings and emissions to 42% by the end of 2023. Thus, the sustainable development indicator decreased to the 2017 level (Figure 2).

The mining, processing, and energy industries—and their related operations—have negative environmental impacts in the areas of deposit development and mineral resource use. In addition to waste generation, the main negative impacts of the mining sector are the deterioration of atmospheric air quality and water pollution.

Part of the industrial waste generated during mining and processing can be sold as a commercial product, returned to the main production process for further extraction of valuable components and production of primary products, or used in the enterprise’s current operations as a raw material, for example, in road construction, backfilling of excavated areas, and dam filling. Some waste-derived resources, such as methane from coal mines, may also be used as fuel, as normally specified in mining and mineral processing design documentation; such materials are classified as recoverable wastes. Reprocessed wastes can be divided into two groups: wastes used as raw materials within the company’s own production process to obtain primary products, and wastes not used for primary production but applied in auxiliary production or other industrial sectors.

The creation of resource-saving technologies combined with environmental protection during mineral resource processing is one of the most urgent tasks of today. The use of industrial wastes in the production of construction composites is a promising way to address this challenge. In recent years, increasing activity has been observed in the use of industrial wastes for producing cement and concrete additives [8,9,10,11,12,13,14,15,16,17,18,19,20].

Therefore, this article considers wastes from technogenic deposits of mining and processing enterprises in the technology of producing mineral modifying additives and, on this basis, heavy, modified, high-performance and high-strength concretes with improved service properties.

The aim of this study is to create and investigate modifying additives based on technogenic mineral raw material wastes new levels of properties, the use of which will ensure the production of a highly workable concrete mixture with increased cohesion and the high-strength concretes based on it.

### Research Gap and Novelty

Studies on the structural modification of cementitious composites are of particular interest because cement is the most widely used binder in the construction industry. One common approach to modifying the structure of cementitious composites is the addition of highly active nanocomponents based on amorphized oxides. Various dispersed and nanodispersed particles, sols, gels, and suspensions are used as concrete additives [8,9,10,11,12,16,17,18,19,20,21,22,23].

The authors of [24] presented a systematic review of modern approaches to improving concrete freeze–thaw resistance using various mineral and chemical modifying additives. Considerable progress has been made in the application of modern materials, including geopolymer concretes and pozzolanic additives, such as metakaolin and silica fume, to increase concrete freeze–thaw resistance. Their work also demonstrated the effects of silica fume, metakaolin, celite, rice-husk ash, graphene oxide, fly ash, and other materials on concrete freeze–thaw resistance up to F300. However, a unified methodology for comparing the results of different studies is lacking, and studies on ternary systems remain limited.

One study [25] examined the effects of silica fume and the water-to-cement ratio on the performance properties of high-strength concrete, including strength, freeze–thaw resistance, water impermeability, water absorption, thermal resistance, and microstructure. The authors noted that improving concrete durability is one of the key challenges of modern construction and that silica fume is an effective means of improving cement stone structure and service performance. At W/C = 0.23, the compressive strength reached 92.85 MPa, water absorption was 0.7%, and freeze–thaw resistance reached 420 cycles; however, at W/C = 0.29, these indicators decreased by up to 30%.

Another study investigated the strength characteristics, durability, and microstructure of pervious concrete modified with 20% fly ash and 10% silica fume. Freeze–thaw resistance of 365–420 cycles and water impermeability grades of W14–W20 were achieved, owing to the low water–cement ratio of 0.23–0.25 and the formation of a dense and homogeneous microstructure with uniform distribution of C-S-H gels [26].

One article [27] focused on the development of an alkali-activated binder based on copper processing waste. In contrast to most previously published studies, where copper tailings were used together with Portland cement, fly ash, blast-furnace slag, or other highly reactive aluminosilicates, the authors investigated, for the first time, the possibility of using copper processing tailings as the sole aluminosilicate component without additional cementitious materials. Thus, the work addressed an urgent problem in modern construction materials science: the creation of low-carbon, cement-free binders using technogenic waste. The results showed that sodium silicate was the most effective activator. The maximum 28-day uniaxial compressive strength reached 16.5 MPa, which was more than twice the strength of specimens activated only with sodium hydroxide (approximately 7.8 MPa), and the freeze–thaw resistance reached 12 cycles.

The authors of [28] compared nano-SiO_2_ synthesized under acidic and alkaline conditions and determined how the synthesis conditions affect nanoparticle dispersion, gel-structure formation, porosity, nanomechanical homogeneity, and geopolymer strength. The key premise of the study is that the effectiveness of nano-SiO_2_ is determined not only by its dosage but also by its dispersion state. Well-dispersed nanoparticles can contribute to the formation of a denser and more continuous geopolymer gel structure, whereas excessive nanomaterial contents lead to agglomeration and reduce matrix homogeneity.

Previous studies on high-performance and durable concretes commonly examine single or binary supplementary cementitious materials, such as silica fume, metakaolin, nanosilica, or recycled mineral fillers, and then evaluate their influence on strength or one selected durability indicator [9,15,19,20,21]. However, less attention has been paid to designing a single modifier that simultaneously combines a highly reactive silica source, mineral processing tailings, and carbonaceous shungite processing waste after mechanochemical activation. This leaves an applied research gap between waste characterization and the development of a performance-oriented concrete modifier.

The contribution of this work is, therefore, not limited to reporting standard concrete tests. The novelty is the formulation of a ternary activated mineral waste modifier, the selection of a 59:40:1 tailings–silica fume–shungite waste ratio based on CaO absorption, and the identification of an 8% dosage that provides a coupled improvement in compressive strength, capillary absorption, freeze–thaw resistance, and water impermeability. This integrated link between raw waste activation, pozzolanic reactivity, dosage optimization, and durability performance is the central scientific and practical outcome of the study.

It was established that the increased durability of concrete containing the complex modifying additive is mainly due to the development of an additional pozzolanic reaction between portlandite formed during cement hydration and the active components of the additive. The following positive effects are achieved: increased formation of secondary calcium silicate hydrate gel (C-S-H); filling of the cement stone pore space, which reduces porosity and increases concrete structural density; and improved adhesion between aggregate and cement stone in the interfacial transition zone.

## 2. Materials and Methods

### 2.1. Materials

The experimental concretes were prepared using CEM I 42.5N Portland cement produced by Shymkentcement LLP (Shymkent, Republic of Kazakhstan); natural sand with a fineness modulus of 2.3, bulk density of 1394 kg/m^3^, and true density of 2.68 g/cm^3^; natural gravel crushed stone with a 5–20 mm fraction, bulk density of 1430 kg/m^3^, and true density of 2.68 g/cm^3^; and high-carbon crushed aggregate obtained from ferrosilicon slag with a 10–20 mm fraction, bulk density of 1492.5 kg/m^3^, and true density of 3.17 g/cm^3^ The chemical composition of the slag aggregate and its X-ray fluorescence results are given in Table 1 and Table 2.

High-carbon crushed aggregate contains Cr(VI) in an amount from 0.1% and may reach approximately 5%. Cr(VI) belongs to hazard class 1 and is considered one of the most dangerous environmental pollutants, posing a serious threat to human health and ecosystems.

The mineral additive was prepared from three components: silica fume from silicon-alloy production, tailings from the Balkhash mining and processing plant, and shungite processing waste from the Koksu deposit. The silica fume contained up to 99.8% SiO_2_, with particle sizes in the approximate range of 100–500 nm. The Balkhash tailings contained SiO_2_, Fe_2_O_3_, Al_2_O_3_, CaO, MgO, and sulfur-bearing phases, and had a fine particle-size distribution. The shungite waste consisted mainly of muscovite–quartz–carbonaceous shale with carbonate minerals. The source-material characteristics are presented in Figure 3, Figure 4, Figure 5 and Figure 6 and Table 3.

Chemical admixtures were used to regulate the rheological and durability characteristics of the cementitious systems. These included Micro Air 800 V as an air-entraining admixture and the superplasticizers Glenium SKY 592 and MasterGlenium ACE 430. Glenium SKY 592 is used for ready-mixed concretes requiring slump retention with variable cements and aggregates, whereas MasterGlenium ACE 430 is used for high early-strength and precast-type mixtures with low water-to-cement ratios.

### 2.2. Methods

The particle-size composition of the mineral modifier was determined by sieving, using No. 09 and No. 008 woven-wire sieves with square openings. Testing was conducted at 20 +3/−2 C and a relative humidity not lower than 50%. Sieving was considered complete when no more than 0.01 g, or 0.1% of the tested material passed through the sieve during control sieving. Tests were performed in accordance with GOST 30744-2001.

Specific surface and density after mechanochemical activation were determined using a Le Chatelier apparatus filled with dehydrated kerosene. The density was calculated as p = m/V, where m is the sample mass and V is the volume of kerosene displaced by the tested material. The reported density and specific surface values were taken as the arithmetic mean of two measurements. Tests were performed in accordance with GOST 30744-2001.

Concrete mixtures were prepared in a 50 L laboratory mixer, with final compositions also checked under production conditions. Workability was evaluated by the Abrams cone slump test. Cubic specimens of 100 × 100 × 100 mm were cast for compressive strength testing and 100 × 100 × 300 mm specimens were prepared for capillary water absorption. Cylindrical specimens 150 mm in diameter and 150 mm in height were used for water-impermeability testing.

Specimens intended for normal curing were stored in molds at 20 ± 5 C before demolding and then cured at 20 ± 2 C and 95 ± 5% relative humidity. Specimens for heat-moisture treatment were cured in a steam chamber according to the adopted regime. Compressive strength was determined at a loading rate of 0.6 ± 0.2 MPa/s and calculated as Rc = alpha(F/A), where F is the failure load, A is the loaded area, and alpha is the scale factor. Tests were performed in accordance with GOST 10180-2012.

Capillary water absorption was evaluated after drying specimens to constant mass at 105 +/− 5 C. The side faces were sealed with a waterproofing composition and the end face was immersed 5–10 mm in water at 20 +/− 5 C. Mass change was used to estimate water uptake. Freeze–thaw resistance was assessed on 100 mm cubes after 28 days of curing using accelerated cycles in a 5% sodium chloride solution. Tests were performed in accordance with GOST 10060-2012.

Water impermeability was determined by the wet-spot method with pressure increased in 0.2 MPa steps and maintained for 16 h at each step. Tests were performed in accordance with GOST 12730.5-2018.

Hexavalent chromium (Cr(VI)) was determined in aqueous extracts of the concrete specimens using 1,5-diphenylcarbazide. The method determines the water-soluble form of Cr(VI). After contact between the specimen and deionized water, the resulting extract was filtered. Sulfuric acid and a 1,5-diphenylcarbazide solution were added to the filtrate and the absorbance of the resulting colored complex was measured at 540 nm using a PE-5400 UV spectrophotometer (Saint Petersburg, Russia). The result for the specimens tested should be reported as follows: Cr(VI) was not detected in the aqueous extract (its concentration was below the detection limit of the method used).

## 3. Results

In this research, the components used as modifiers were activated. Mechanical activation in different mills is one of the most common operations in mechanochemistry because of its relative simplicity and because milling is a widely used method of mechanical action on raw materials. Grinding was performed to obtain the maximum modifier surface area at minimum energy consumption and to activate the material through changes in the solid phase.

Mechanochemical activation of the components for producing the modifier was carried out in a ball mill using cylpebs and balls as grinding media. The grinding media characteristics were as follows: balls, d = 4.0, mass of one ball = 325–430 g; cylpebs, length = 2, d = 2, and mass = 50 g. The grinding duration ranged from 30 min to 60 min. The modifier characteristics were monitored using sieve analysis and determination of the specific surface area.

The dependence of the dispersion degree of the ground modifier on grinding time and grinding media type is presented in Table 4.

The data in Table 4 show that steel cylpebs grind the material more effectively than steel balls. The fineness determined by residue on the 008 sieve differed by 1.9 +/− 0.1% after 30 min of grinding, by 1.8 +/− 0.1% after 45 min, and by 1.9 +/− 0.1% after 1 h. The results also indicate that 30–45 min of grinding is sufficient to activate the surface of the modifier components, because further grinding does not substantially change the sieve residues; the difference is only 0.2%, regardless of the grinding media type. When a solid body is fractured, new surfaces are formed, on which the physical phenomenon of fast-electron emission occurs in several stages: surface electrification during fracture of the solid; electron-emission-related effects; formation of free radicals; and release of gaseous substances.

Thus, the results indicate that grinding of the raw components with cylpebs is more effective than grinding with balls; the grinding duration in the ball mill can be reduced by using cylpebs as the grinding media; and loading the ball mill with grinding media at 40–60% improves abrasion.

The specific surface area of the obtained modifiers ranged from 3100 to 3580 cm^2^/g.

Pozzolanic activity was determined using a simplified Frattini-type procedure. One gram of additive was mixed with 75 mL of saturated lime solution and held in sealed flasks at 40 C for 3, 7, and 28 days. The filtrate was titrated for OH^−^ and Ca^2+^ content. The mean relative error ranged from 2% to 5%.

The compositions and 28-day CaO absorption values are summarized in Table 5.

The data in the table show that mineral processing tailings have low pozzolanic activity; however, the presence of silica fume substantially changes this value, while shungite nanowaste has a minor additional effect. This process is supported by other authors’ findings: lime absorption by finely ground silica occurs in two periods. The first period corresponds to a sharp initial change in calcium hydroxide concentration caused by Ca(OH)_2_ adsorption on SiO_2_ particles. The second period is characterized by a lower reaction rate and a continuing decrease in concentration due to the precipitation of calcium silicate hydrate formed by the chemical reaction.

Therefore, a mineral additive consisting of mineral processing tailings, silica fume, and shungite waste, at a ratio of 59:40:1, was selected for further investigation; according to the relevant regulatory document, this additive belongs to the medium pozzolanic activity category.

Using empirical selection based on mathematical methods of experimental design, high-strength, high-performance B40 concretes were developed and tested for compressive strength, freeze–thaw resistance, and water impermeability. Accelerated capillary suction tests were also conducted to determine capillary porosity as a function of freeze–thaw resistance; temperature–moisture deformation tests were performed to establish the relationship between freeze–thaw resistance and reduced elongation during freezing and thawing.

The control B40 concrete mixture (compressive strength not less than 52.39 MPa) subjected to standard moist curing had the following composition by mass: Portland cement, 530 kg/m^3^; coarse aggregate (55% natural crushed stone and 45% high-carbon slag crushed stone), 905 kg/m^3^; natural sand, 830 kg/m^3^; Glenium SKY 592 superplasticizer, 3.64 kg/m^3^ (0.7% by mass of cement); Micro Air 800 V, 1.04 kg/m^3^ (0.2% by mass of cement); and water, 159.6 L/m^3^. The mean compressive strength was 34.58 MPa at 7 days and 52.41 MPa at 28 days. The w/c ratio was 0.30.

The modified B40 concrete mixtures subjected to standard moist curing had the same composition as the control mixture, except that the mineral modifier replaced 3% (15.9 kg/m^3^), 5% (26.5 kg/m^3^), 8% (42.4 kg/m^3^), 10% (53 kg/m^3^), 15% (79.5 kg/m^3^), or 20% (106 kg/m^3^) of the cement by mass, with no change in the chemical admixture dosages. The water content ranged from 161.5 to 164.2 L/m^3^. The w/b ratio remained 0.30. The mixture proportions for B40 concrete subjected to standard moist curing are presented in Table 6.

**Table 6:** Mixture proportions for B40 concrete subjected to standard moist curing.

Constituent	Mixture Number and Component Quantity, kg/m^3^					
	No. 1	No. 2	No. 3	No. 4	No. 5	No. 6
Portland cement	514.1	503.5	487.6	477	450.5	424
Coarse aggregate	905	905	905	905	905	905
Fine aggregate	830	830	830	830	830	830
Mineral modifier	15.9	26.5	42.4	53	79.5	106
Glenium SKY 592 superplasticizer	3.64	3.64	3.64	3.64	3.64	3.64
Micro Air 800 V	1.04	1.04	1.04	1.04	1.04	1.04
Water	161.5	162.0	162.6	163.2	163.9	164.2

The control B40 concrete mixture (compressive strength not less than 52.39 MPa) subjected to heat-moisture treatment had the following composition by mass: Portland cement, 530 kg/m^3^; coarse aggregate (55% natural crushed stone and 45% high-carbon slag crushed stone), 905 kg/m^3^; natural sand, 830 kg/m^3^; MasterGlenium ACE 430 superplasticizer, 3.64 kg/m^3^ (0.7% by mass of cement); Micro Air 800 V, 1.04 kg/m^3^ (0.2% by mass of cement); and water, 160.8 L/m^3^. The compressive strength was 36.02 MPa after heat-moisture treatment and 52.40 MPa at 28 days.

The modified B40 concrete mixtures subjected to heat-moisture treatment had the same composition as the control mixture, except that the mineral modifier replaced 3% (15.9 kg/m^3^), 5% (26.5 kg/m^3^), 8% (42.4 kg/m^3^), 10% (53 kg/m^3^), 15% (79.5 kg/m^3^), or 20% (106 kg/m^3^) of the cement by mass, with no change in the chemical admixture dosages. The water content ranged from 162.3 to 165.7 L/m^3^, and the w/b ratio ranged from 0.30 to 0.31. The mixture proportions for B40 concrete subjected to heat-moisture treatment are presented in Table 7.

**Table 7:** Mixture proportions for B40 concrete subjected to heat-moisture treatment.

Constituent	Mixture Number and Component Quantity, kg/m^3^					
	No. 1	No. 2	No. 3	No. 4	No. 5	No. 6
Portland cement	514.1	503.5	487.6	477	450.5	424
Coarse aggregate	905	905	905	905	905	905
Fine aggregate	830	830	830	830	830	830
Mineral modifier	15.9	26.5	42.4	53	79.5	106
MasterGlenium ACE 430 superplasticizer	3.64	3.64	3.64	3.64	3.64	3.64
Micro Air 800 V	1.04	1.04	1.04	1.04	1.04	1.04
Water	162.3	162.7	163.3	164.2	164.9	165.7

The concrete mixtures had a slump of 23–25 cm, corresponding to slump grade P5.

The compressive strength results for concretes cured under normal conditions and after heat-moisture treatment at 70 °C, at ages of 7 and 28 days, are presented in Figure 7. Three series of specimens of the same composition were tested, with 12 cubes measuring 100 × 100 × 100 mm in each series.

The compressive strength for each specimen series was calculated as the arithmetic mean of the strengths of the tested specimens in that series.

- For three specimens cast in a three-gang mold, the two specimens with the highest strengths were used.

Thus, of the results obtained for 12 cube specimens, eight values were used to calculate the mean for each set. With three series, this yielded a total of 24 values.

For cube specimens subjected to standard moist curing, the 7-day compressive strength ranges were as follows: Mixture No. 1, 33.83–39.47 +/− 0.2 MPa; Mixture No. 2, 41.10–47.56 +/− 0.2 MPa; Mixture No. 3, 43.02–49.19 +/− 0.2 MPa; Mixture No. 4, 42.30–48.90 +/− 0.2 MPa; Mixture No. 5, 32.49–34.83 +/− 0.2 MPa; and Mixture No. 6, 32.20–34.0 +/− 0.2 MPa. At 28 days, the corresponding ranges were as follows: Mixture No. 1, 52.93–58.49 +/− 0.2 MPa; Mixture No. 2, 53.32–58.03 +/− 0.2 MPa; Mixture No. 3, 56.23–63.45 +/− 0.2 MPa; Mixture No. 4, 53.95–60.09 +/− 0.2 MPa; Mixture No. 5, 50.88–55.27 +/− 0.2 MPa; and Mixture No. 6, 49.79–52.60 +/− 0.2 MPa.

For cube specimens subjected to heat-moisture treatment, the compressive strength ranges immediately after treatment were as follows: Mixture No. 1, 35.11–40.12 +/− 0.2 MPa; Mixture No. 2, 39.49–44.80 +/− 0.2 MPa; Mixture No. 3, 44.56–50.23 +/− 0.2 MPa; Mixture No. 4, 40.68–47.15 +/− 0.2 MPa; Mixture No. 5, 32.92–37.30 +/− 0.2 MPa; and Mixture No. 6, 32.98–36.96 +/− 0.2 MPa. At 28 days, the corresponding ranges were as follows: Mixture No. 1, 52.13–59.16 +/− 0.2 MPa; Mixture No. 2, 53.45–60.21 +/− 0.2 MPa; Mixture No. 3, 58.10–65.31 +/− 0.2 MPa; Mixture No. 4, 54.13–59.37 +/− 0.2 MPa; Mixture No. 5, 50.16–54.05 +/− 0.2 MPa; and Mixture No. 6, 49.14–53.07 +/− 0.2 MPa.

The graph presents the mean compressive strength of cube specimens. Mixture No. 1 had values of 36.68, 55.40, 36.15, and 56.90 +/− 0.2 MPa with 3% mineral modifying additive; Mixture No. 2 had values of 42.15, 56.21, 43.64, and 55.90 +/− 0.2 MPa with 5%; Mixture No. 3 had values of 46.20, 60.84, 45.82, and 59.63 +/− 0.2 MPa with 8%; Mixture No. 4 had values of 43.24, 56.92, 45.34, and 57.40 +/− 0.2 MPa with 10%; Mixture No. 5 had values of 35.10, 52.53, 34.24, and 53.73 +/− 0.2 MPa with 15%; and Mixture No. 6 had values of 35.02, 52.40, 33.62, and 51.44 +/− 0.2 MPa with 20%. Analysis of these data shows that the strength increase was 5.7–8.5% for Mixture No. 1, 6.6–7.2% for Mixture No. 2, 13.7–16.0% for Mixture No. 3, 8.5–9.5% for Mixture No. 4, and 0.19–2.48% for Mixture No. 5. The greatest increase was obtained for Mixture No. 3 containing 8% mineral modifying additive, whereas Mixture No. 6 showed a strength decrease of up to 1.9%.

Early-age strength was evaluated for mixtures containing Micro Air 800 V, Glenium SKY 592, MasterGlenium ACE 430, and the mineral modifying additive. After one day of normal moist curing, concretes with the mineral modifying additive reached 17.0–32.3 MPa, with the greatest early-strength gains observed for mixtures No. 2, No. 3, and No. 4 (Figure 8).

Concrete strength class, freeze–thaw resistance grade, and water-impermeability grade should not be considered independently. Because all concrete properties are determined by its structure, which depends mainly on the water–cement ratio, relationships between these performance indicators exist but are highly non-linear.

Available research data show that there is no direct relationship between compressive strength and concrete freeze–thaw resistance, especially when air is entrained in concrete. The method and type of air entrainment substantially affect the nature of conditionally closed porosity, which is known to have a major influence on freeze–thaw resistance.

To assess the freeze–thaw resistance of high-performance concretes, the capillary suction method, also known as capillary water absorption, was applied. This method is not used for the direct measurement of freeze–thaw resistance but for its indirect assessment, because freeze–thaw resistance is closely related to concrete permeability and pore structure. Capillary water absorption was determined by measuring the volume of water absorbed by a specimen dried to constant mass under atmospheric pressure through capillary or adsorption forces.

Capillary water absorption was used as an indirect durability indicator because frost resistance is strongly influenced by permeability and pore structure. The 24 h results showed the largest increase in capillary uptake, after which the change at 48 h was comparatively small. This supports the use of 24 h capillary absorption as a practical indicator for assessing potential freeze–thaw resistance The measured values are reported in Table 8.

According to Table 8, the changes in capillary suction over the specified time intervals were as follows: Mixture No. 1, 0.72-2.63-1.47-0.50%; Mixture No. 2, 0.92-2.24-1.14-0.78%; Mixture No. 3, 0.53-2.54-1.06-0.17%; Mixture No. 4, 0.54-2.44-1.28-0.16%; Mixture No. 5, 1.03-2.76-1.24-0.63%; and Mixture No. 6, 1.12-2.98-1.07-0.66%. Three series of specimens of the same composition were tested, with three prism specimens measuring 300 × 100 × 100 mm in each series.

Thus, the obtained data show that the maximum increase in capillary suction occurs after 24 h, whereas, after 48 h, the increase ranges from 0.16% to 0.78%. Therefore, capillary suction can be assessed after 24 h and used as an indirect indicator of concrete freeze–thaw resistance.

The relationship between concrete capillary suction and freeze–thaw resistance is based on the principle that the faster concrete absorbs water, the faster it reaches critical saturation (S approximately 0.9), upon which concrete damage begins to occur under negative temperatures.

Capillary suction is caused by liquid surface tension and the wettability of cement stone pore surfaces. Water penetrates into concrete through an interconnected system of capillary pores, filling them according to their size and structure. The intensity of capillary water absorption is directly related to total material porosity, capillary pore radius, degree of pore connectivity, and cement stone microstructure. The higher the proportion of open capillary pores, the faster concrete becomes saturated with water, which increases the risk of low freeze–thaw resistance. Intensive water absorption indicates a developed capillary pore system; such concrete is characterized by a high degree of water saturation, and, consequently, lower freeze–thaw resistance. At lower absorption coefficients, the concrete structure is denser and has limited capillary permeability, which reduces water saturation and improves freeze–thaw resistance.

## 4. Discussion

The improvement in compressive strength and durability can be attributed to the combined action of particle packing, pozzolanic reactions, and modification of the cement stone microstructure. Fine silica-rich particles consume calcium hydroxide and promote the formation of additional calcium silicate hydrate phases. Mechanochemical activation increases the specific surface area and creates more reactive surfaces, which can accelerate hydration and densify the interfacial transition zone [16,17,18,19,22,23,24,25,26,27].

The main novelty of the obtained results is the coupled nature of the performance optimum. Not only did the 8% dosage increase compressive strength, it also coincided with improved capillary water behavior, F400 freeze–thaw resistance, and W14 water impermeability. This indicates that the modifier affected the pore system and cement stone structure in a way that cannot be fully described by strength gain alone.

This distinguishes the proposed additive from the conventional use of silica fume or inert mineral fillers, where the material is often introduced as a single replacement component and assessed mainly by strength. In the present work, the activated ternary composition is treated as a multifunctional modifier: it combines reactive silica, mineral waste fines, and shungite-derived material, and its dosage is optimized against both mechanical and transport-related durability indicators.

It has been established that, in studies of concrete freeze–thaw resistance in saline solution, the specific surface area of air pores characterizes freeze–thaw resistance more accurately [8,9,10,11,12,13,14,15,24,25,26]. High freeze–thaw resistance was observed when the specific surface area of pores exceeded 1000 m^2^/m^3^. This result can be explained as follows: concrete damage occurs when water freezes in capillaries, whereas damage does not occur when water freezes in air pores, where a certain volume of air remains. As the specific surface area of pores increases, more capillaries are connected to air pores and fewer capillaries remain without such outlets, ultimately improving concrete freeze–thaw resistance.

Additives with particle sizes from 1 to 100 µm can also fill larger capillary pores and participate in the pozzolanic reaction. This leads to the formation of additional calcium silicate hydrates, changes the total porosity, and makes the structure denser.

The authors of [11] established that freeze–thaw resistance is determined not only by total porosity but also by pore-size distribution. The most favorable structure contained the maximum number of pores smaller than 100 nm. Such pores practically do not participate in ice accumulation and do not generate dangerous internal stresses.

Freeze–thaw durability is governed not only by compressive strength but also by pore connectivity, air-void structure, and the ability of concrete to avoid critical saturation. The use of complex modifiers, together with air-entraining admixtures, changes the parameters of conditionally closed porosity, increases the concentration of smaller pores, and reduces the spacing factor. However, the spacing factor alone is insufficient for assigning a frost-resistance grade; thus, direct freeze–thaw testing was performed. The direct freeze–thaw results are summarized in Table 9. Three series of specimens of the same composition were tested, with 15 cubes measuring 100 × 100 × 100 mm in each series.

Mixtures No. 1, No. 2, No. 5, and No. 6 withstood 75 freeze–thaw cycles and corresponded to grade F300. Mixtures No. 3 and No. 4 withstood 110 cycles and corresponded to grade F400. The evaluation considered three criteria: average mass loss not exceeding 2%, satisfaction of the compressive strength confidence criterion XminII >= 0.9 XminI, and absence of cracks, spalling, or edge-scaling. The water-impermeability results obtained using the wet-spot method are presented in Table 10.

**Table 10:** Water impermeability results for concretes tested by the wet-spot method.

Mixture No.	Water Pressure Resisted MPa	Water-Impermeability Grade
Mixture No. 1	1.0	W10
Mixture No. 2	1.2	W12
Mixture No. 3	1.4	W14
Mixture No. 4	1.4	W14
Mixture No. 5	1.2	W12
Mixture No. 6	0.8	W8

The developed concretes also showed high water impermeability, reaching a resisted water pressure of 1.4 MPa, corresponding to grade W14. This performance is consistent with the lower capillary absorption and the denser microstructure produced by the combined mineral and chemical admixture system. The practical importance of this result is that the same activated waste-derived modifier produced a coherent durability response across water uptake, freeze–thaw resistance, and water pressure resistance, which is more relevant for monolithic and infrastructure concretes than an isolated strength increase. Three series of specimens of the same composition were tested, with six cylindrical specimens measuring 150 × 150 mm in each series.

Studies to determine Cr(VI) in the aqueous extract showed its absence due to the dense concrete structure and high water impermeability, which effectively immobilized and encapsulated Cr(VI) within the concrete body.

Scanning electron microscopy of cement stone cured under normal moist conditions for 28 days showed that the introduction of the mineral modifying additive led to the appearance of gel-like regions, with a CaO/SiO_2_ ratio of 2.9–3.0, locally covering cement grains (Figure 9). A similar pattern was observed after heat-moisture treatment, although the gel-like regions occurred slightly less frequently and had a CaO/SiO_2_ ratio of 2.6–2.8. The effect of the mineral modifying additive is expressed as a certain increase in the basicity of the formed calcium silicate hydrates due to the presence of gel-like regions of a metastable mother liquor supersaturated with respect to CaO and the appearance of crystallization centers, indicating intensified hydration processes in cement stone.

The authors of [25] paid particular attention to the effect of silica fume on concrete microstructure. Scanning electron microscopy showed the formation of a significant amount of secondary calcium silicate hydrate (C-S-H), which forms as a result of the pozzolanic reaction between silica fume and calcium hydroxide. Formation of additional C-S-H gel densifies the cement stone structure, reduces capillary pore size, increases the density of the interfacial transition zone between cement stone and aggregate, and decreases concrete permeability. These processes are the main reason for the improved strength, freeze–thaw resistance, and water impermeability of the studied mixtures.

**Figure 9:**
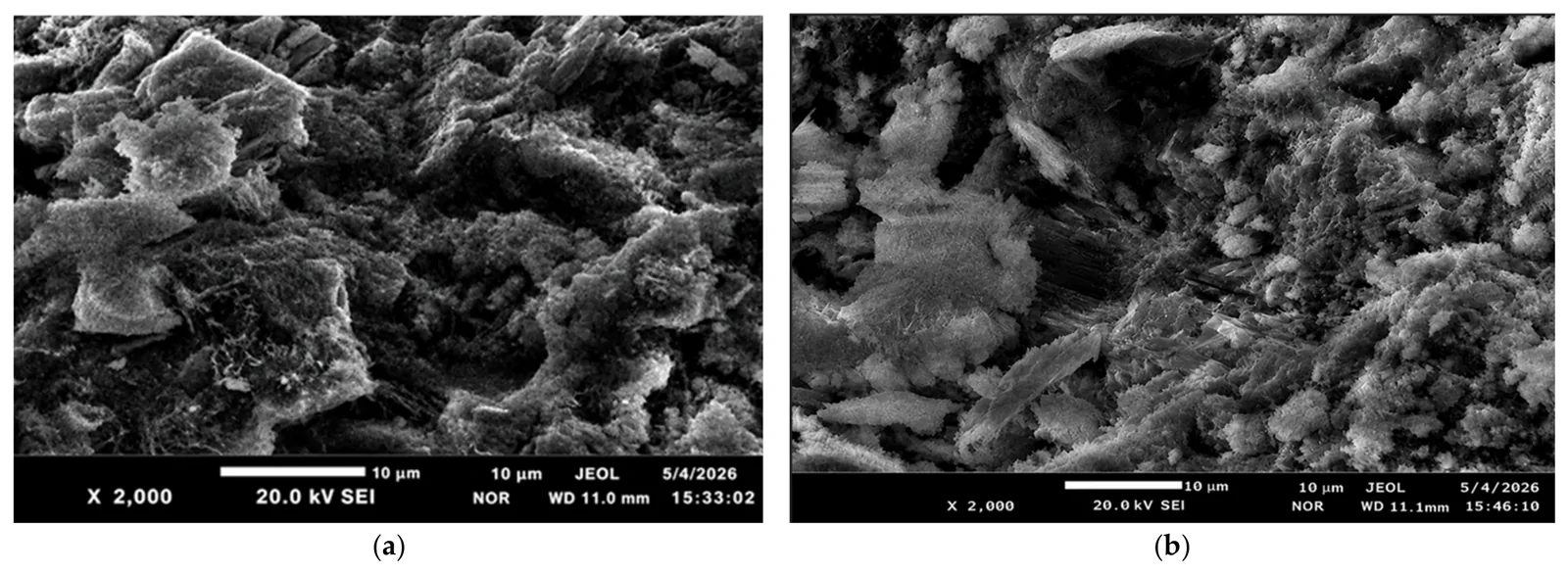
(**a**). cement stone microscopy for concrete with 8% mineral additive under normal moist curing; and (**b**). cement stone microscopy for concrete with 8% mineral additive after heat-moisture treatment.

The X-ray diffraction pattern (Figure 10) for cement stone hardened in the presence of 8% complex mineral additive showed lines of tricalcium silicate C_3_S (3.037, 2.76, 2.32, 2.188, 1.853, 1.762, and 1.48 Å), dicalcium silicate beta-C_2_S (2.89 Å), quartz (4.27, 3.34, 2.28, 1.821, and 1.539 Å), and the hydration products Ca(OH)_2_ (4.917, 2.625, 1.924, 1.793, and 1.684 Å), CaCO_3_ (3.037, 2.28, and 2.093 Å), and ettringite (9.73, 3.871, 2.77, and 2.18 Å). On the differential thermal curve, endothermic effects at 120 °C and 150 °C were associated with ettringite dehydration and transition to CaO, the effect at 520 °C was associated with Ca(OH)_2_ dehydration, and the effect at 800 °C was associated with calcite decomposition.

According to the X-ray phase analysis, the mineral additive had a positive effect on clinker-mineral hydration, causing an increase in the intensity of the Ca(OH)_2_ line with an interplanar spacing of 4.92 Å and the appearance of an additional Ca(OH)_2_ line at 2.63 Å. Endothermic effects at 50 °C and 520 °C, associated with water removal from Ca(OH)_2_, were recorded on the thermograms.

Thermogravimetric analysis of cement stone with mineral additives confirmed the X-ray analysis results regarding the effect of the additives on hydration processes. The degree of cement hydration, determined by mass loss after 28 days, reached 23%.

The measurements were performed at JSC Institute of Metallurgy and Ore Beneficiation (Almaty, Republic of Kazakhstan) using a D8 Advance instrument (Bruker), Cu-alpha radiation, tube voltage of 40 kV, and current of 40 mA. Diffraction data processing and calculation of interplanar spacings were performed using EVA software (https://www.bruker.com/en/products-and-solutions/diffractometers-and-scattering-systems/x-ray-diffractometers/diffrac-suite-software/diffrac-eva.html, accessed on 25 September 2026). Phase identification and sample interpretation were carried out using Search/Match software (https://www.icdd.com/pdf-2/, accessed on 25 September 2026) with the PDF-2 powder diffraction database.

**Figure 10:**
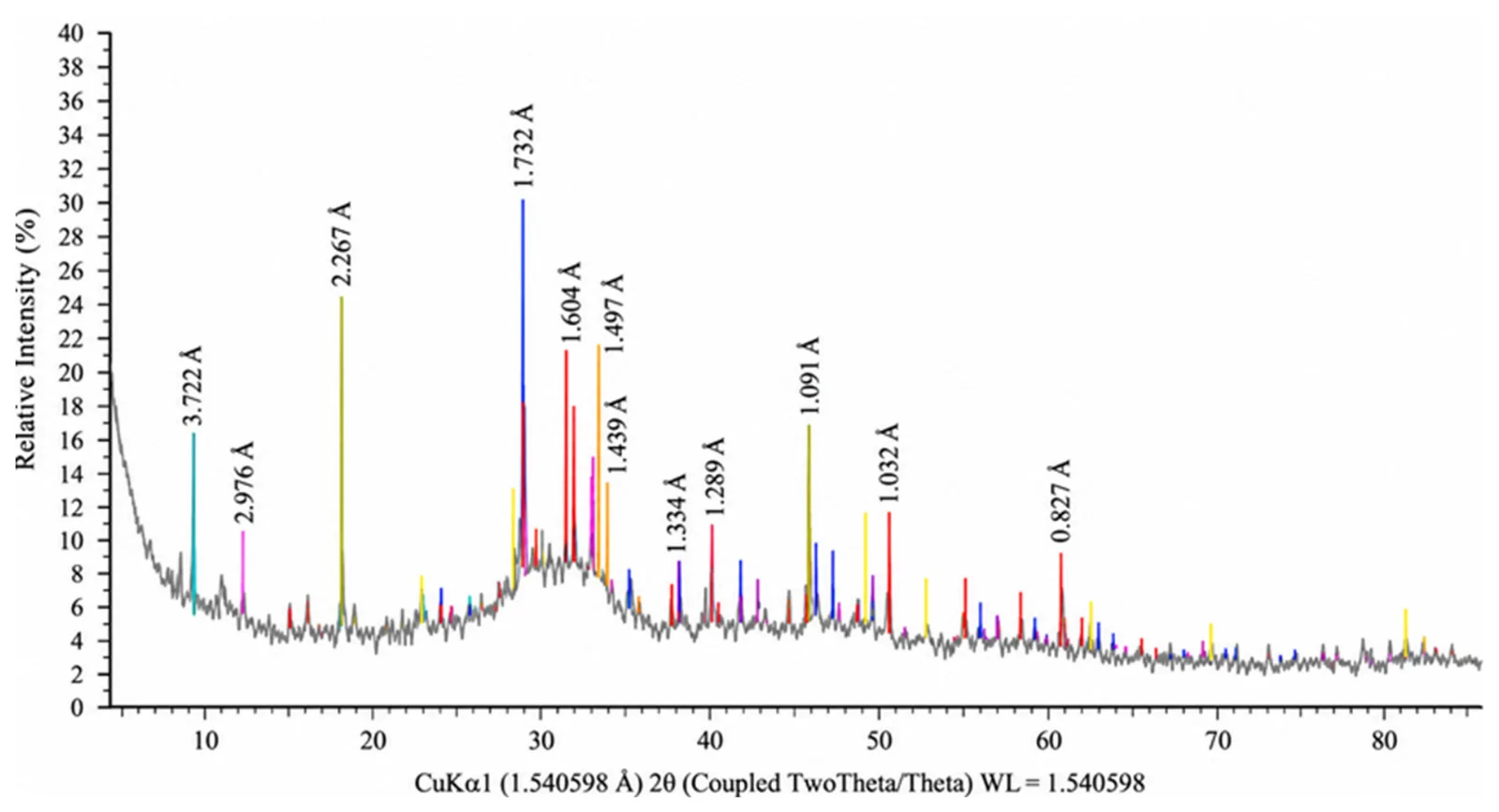
X-ray diffraction pattern of processes occurring in the cement-complex mineral additive system (8%).

## 5. Conclusions

The results of the conducted research make it possible to formulate the following main conclusions.

1. During mechanochemical activation of the mineral modifying additive, it was established that grinding media in the form of steel cylpebs grind more effectively than steel balls. Fineness, determined by residue on the 008 sieve, was 90.6% and 92.5% after 30 min, and 90.9% and 92.7% after 45 min of grinding. When a solid body is fractured, new surfaces are formed, on which the physical phenomenon of fast-electron emission occurs in several stages: surface electrification during fracture of the solid; electron emission-related effects; formation of free radicals; and release of gaseous substances. The specific surface area of the obtained mineral modifiers ranged from 3100 to 3580 cm^2^/g;

2. The mineral modifying additive consisting of mineral processing tailings, silica fume, and shungite waste at a ratio of 59:40:1 showed pozzolanic activity corresponding to 59.2% CaO absorption after 28 days. Mineral processing tailings have low pozzolanic activity; however, silica fume significantly changes this value, and shungite nanowaste also contributes to it. Specifically, lime absorption by finely ground silica occurs in two stages. The first stage corresponds to a sharp initial change in calcium hydroxide concentration caused by Ca(OH)_2_ adsorption on SiO_2_ particles. The second stage is characterized by a lower rate and a continuing decrease in concentration due to precipitation of calcium silicate hydrate formed by the chemical reaction;

3. The compressive strength of concretes based on the mineral modifying additive was determined at 7 and 28 days under normal moist curing and after heat-moisture treatment at 70 °C. The data show compressive strength increases of 5.7–8.5% for Mixture No. 1, 6.6–7.2% for Mixture No. 2, 13.7–16.0% for Mixture No. 3, 8.5–9.5% for Mixture No. 4, and 0.19–2.48% for Mixture No. 5. The greatest increase was obtained for Mixture No. 3, containing 8% mineral modifying additive. One-day compressive strength tests under normal moist curing with the mineral modifying additive showed values from 17.0 to 32.3 MPa. Among all mixtures, the greatest early-strength increase was observed for Mixtures No. 2, No. 3, and No. 4, containing 3%, 5%, and 8% mineral additive, respectively;

4. The capillary-suction method showed that after 24 h, the greatest increase in capillary suction ranged from 4.13% to 5.17%, whereas after 48 h, the increase ranged from 0.16% to 0.78%. Therefore, capillary suction can be assessed after 24 h and used as an indirect indicator of concrete freeze–thaw resistance. Capillary suction is caused by liquid surface tension and the wettability of cement stone pore surfaces. The intensity of capillary water absorption is directly related to total material porosity, capillary pore radius, degree of pore connectivity, and cement stone microstructure. The higher the proportion of open capillary pores, the faster the concrete becomes saturated with water, which increases the risk of low freeze–thaw resistance;

5. The combined introduction of complex modifiers and air-entraining additives was found to change the parameters of conditionally closed concrete porosity, increase the concentration of fine pores, and reduce the spacing factor. Along with the spacing factor, the total pore volume and the number of pores per unit volume play an important role. The concretes were subjected to alternate freezing in a 5% aqueous sodium chloride solution at −18 °C and thawing in a 5% aqueous sodium chloride solution at +20 °C. Mixtures No. 1, No. 2, No. 5, and No. 6 withstood 75 freezing–thawing cycles, corresponding to freeze–thaw resistance grade F300, whereas Mixtures No. 3 and No. 4 withstood 110 cycles, corresponding to grade F400. Three main criteria were considered when assigning the freeze–thaw resistance grade: average mass loss not exceeding 2%, compliance with the compressive strength ratio XminII >= 0.9 XminI, and absence of cracks, spalling, and edge-scaling on the specimens;

6. The water impermeability of concretes based on the mineral additive was determined, and the concretes showed high-performance values reaching 1.4 MPa, corresponding to grade W14. This result was influenced by the combined action of the mineral modifying additive and chemical additives. High water impermeability and the dense concrete structure contribute to the immobilization and encapsulation of Cr(VI) within the concrete body and prevent negative impacts on the ecosystem;

7. The increased durability of concrete containing the complex modifying additive is mainly due to the development of an additional pozzolanic reaction between portlandite formed during cement hydration and the active components of the additive. As a result of the processes recorded by XRD, DTA, and electron microscopy, the following positive effects are achieved: increased formation of secondary calcium silicate hydrate (C-S-H) gel; filling of the pore space of cement stone, which reduces porosity and increases the density of the concrete structure; and improved adhesion between aggregate and cement stone in the interfacial transition zone.

## Figures and Tables

**Figure 1:**
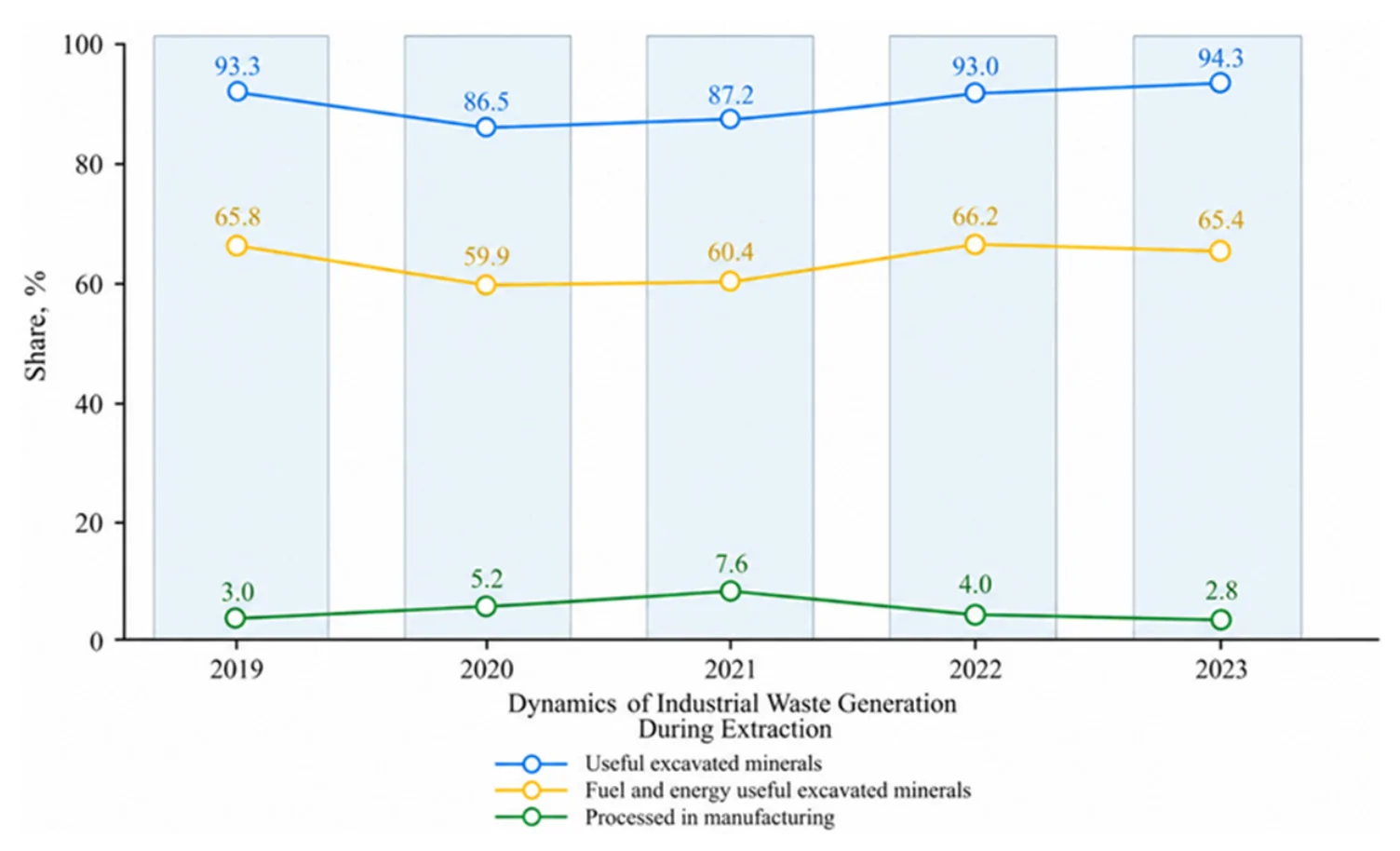
Dynamics of industrial waste generation in Kazakhstan.

**Figure 2:**
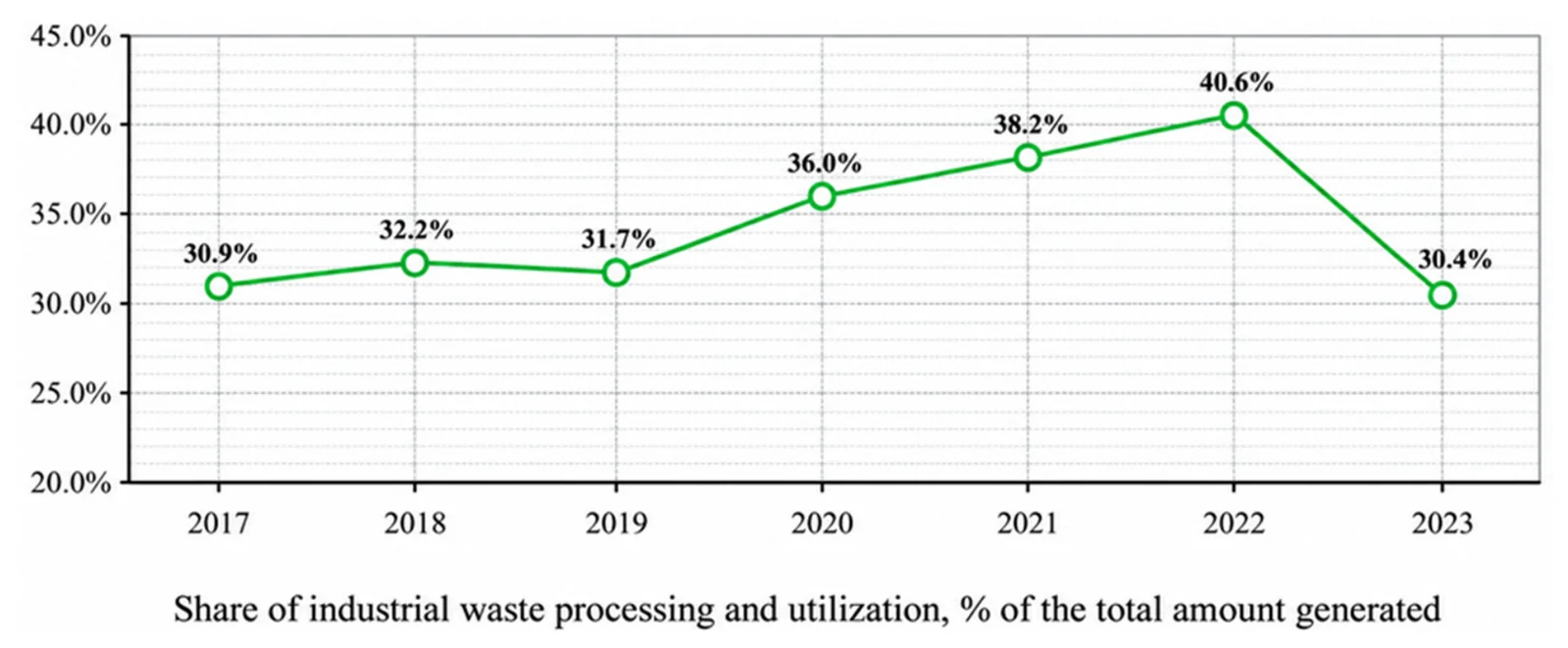
Share of industrial waste recycling and utilization.

**Figure 3:**
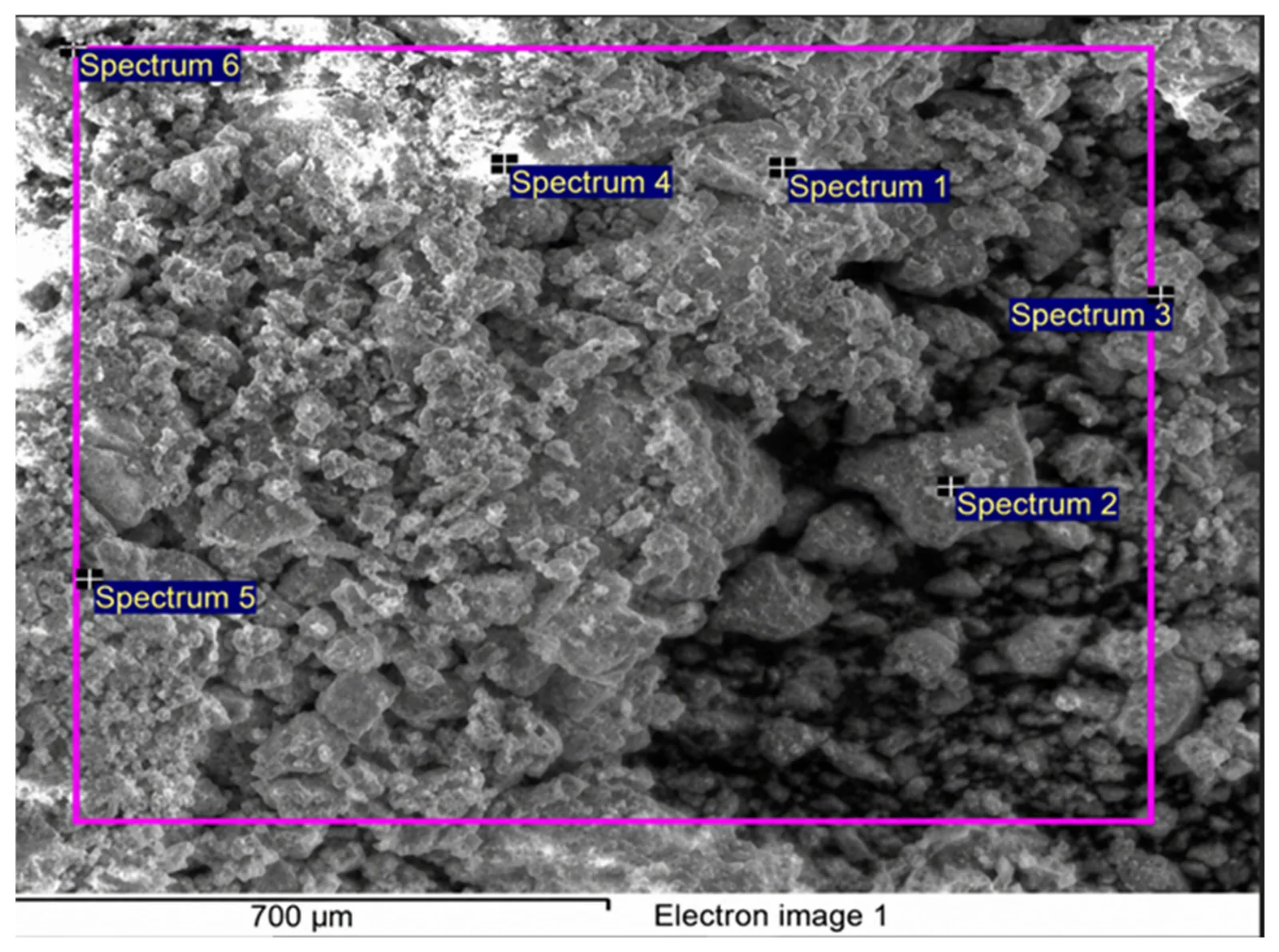
EDS spectra of silica fume supplied by General Service.

**Figure 4:**
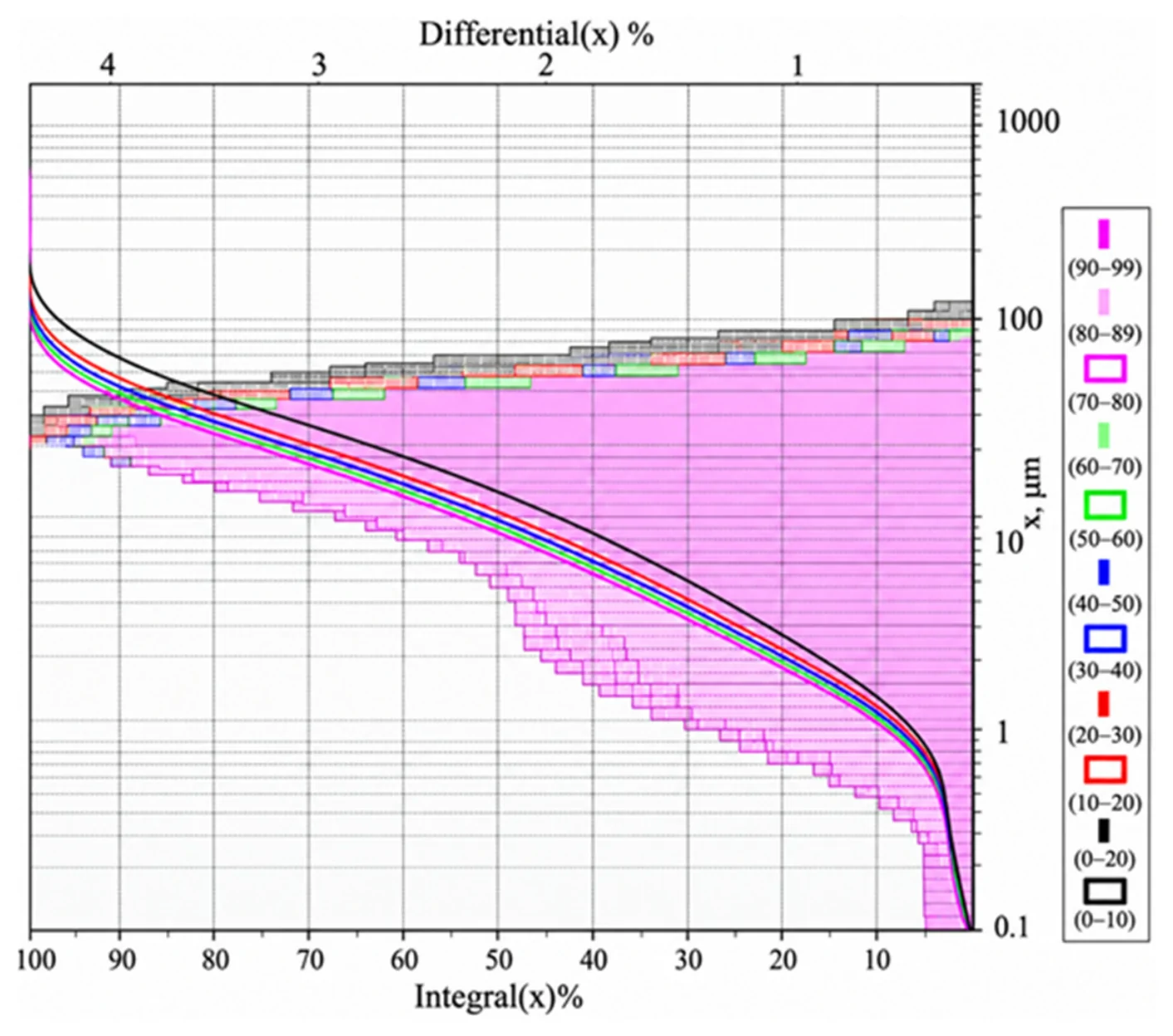
Particle-size distribution of tailings from the Balkhash mining and processing plant.

**Figure 5:**
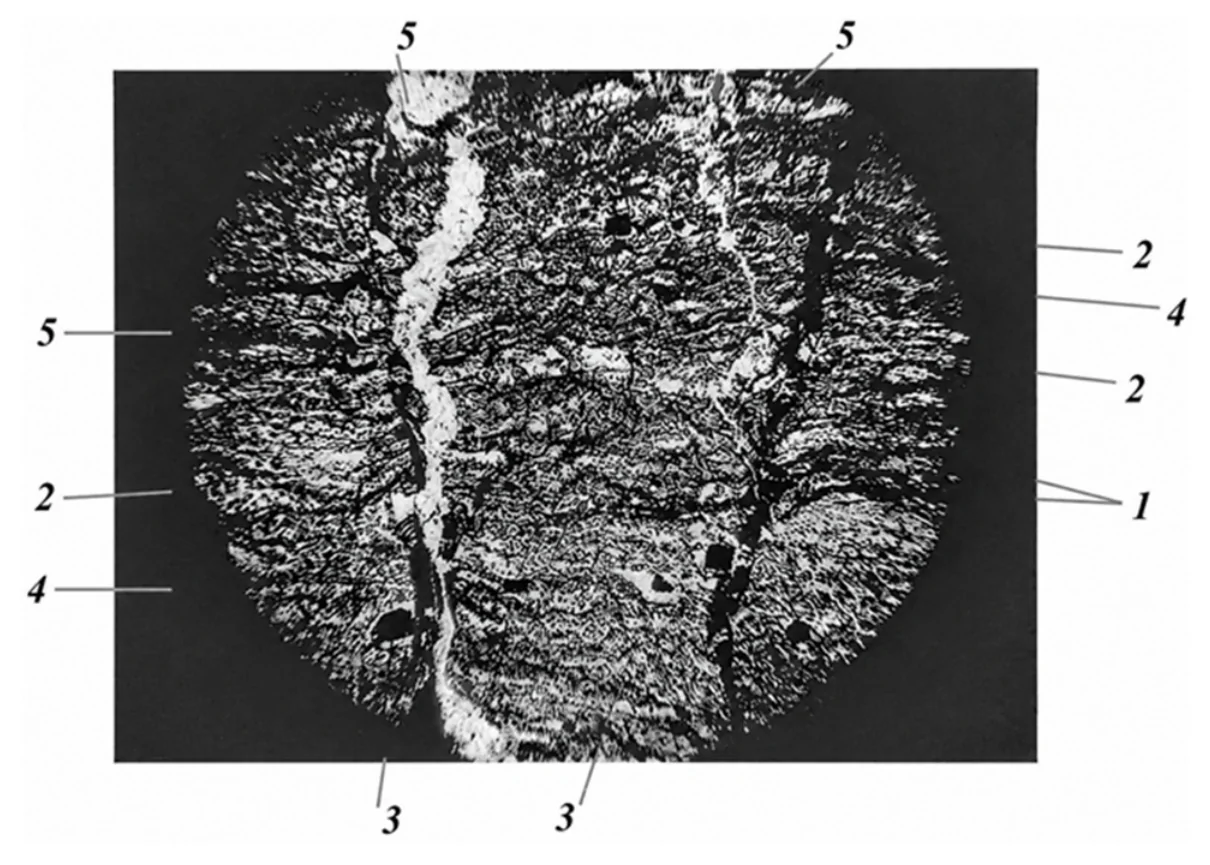
Thin section of shungite sample 2 at 50× magnification.

**Figure 6:**
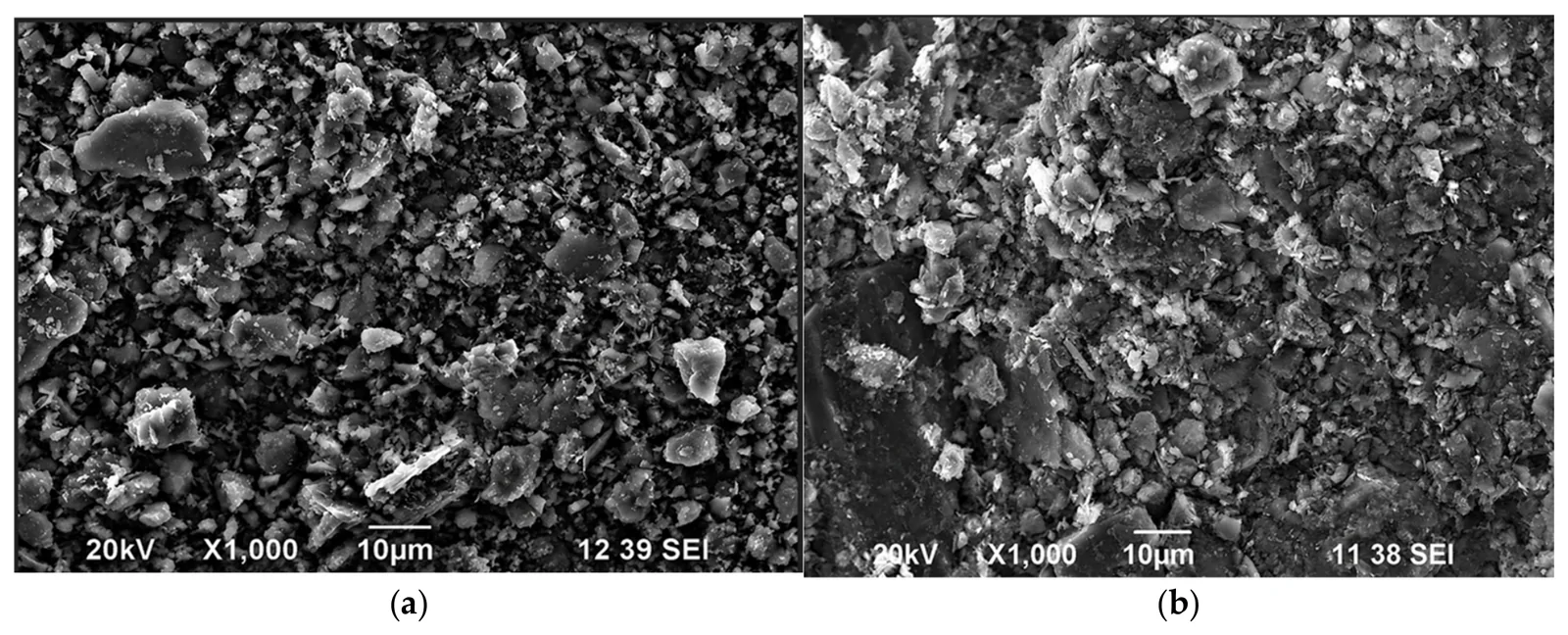
(**a**). microstructure of the 20 mm shungite fraction; and (**b**) microstructure of the 1 mm shungite fraction.

**Figure 7:**
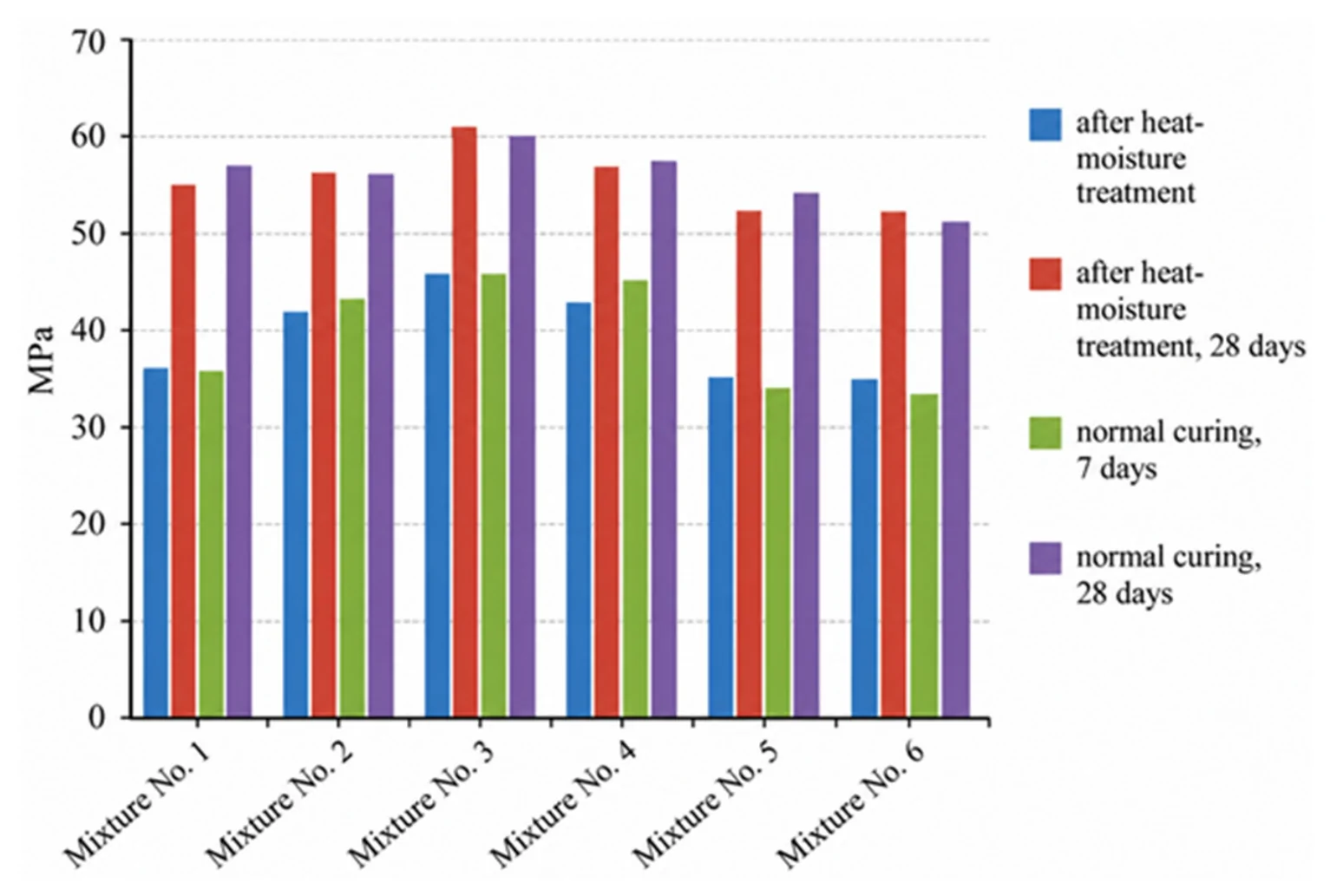
Influence of mineral modifying additive dosage on compressive strength of B40 concrete under normal curing and heat-moisture treatment.

**Figure 8:**
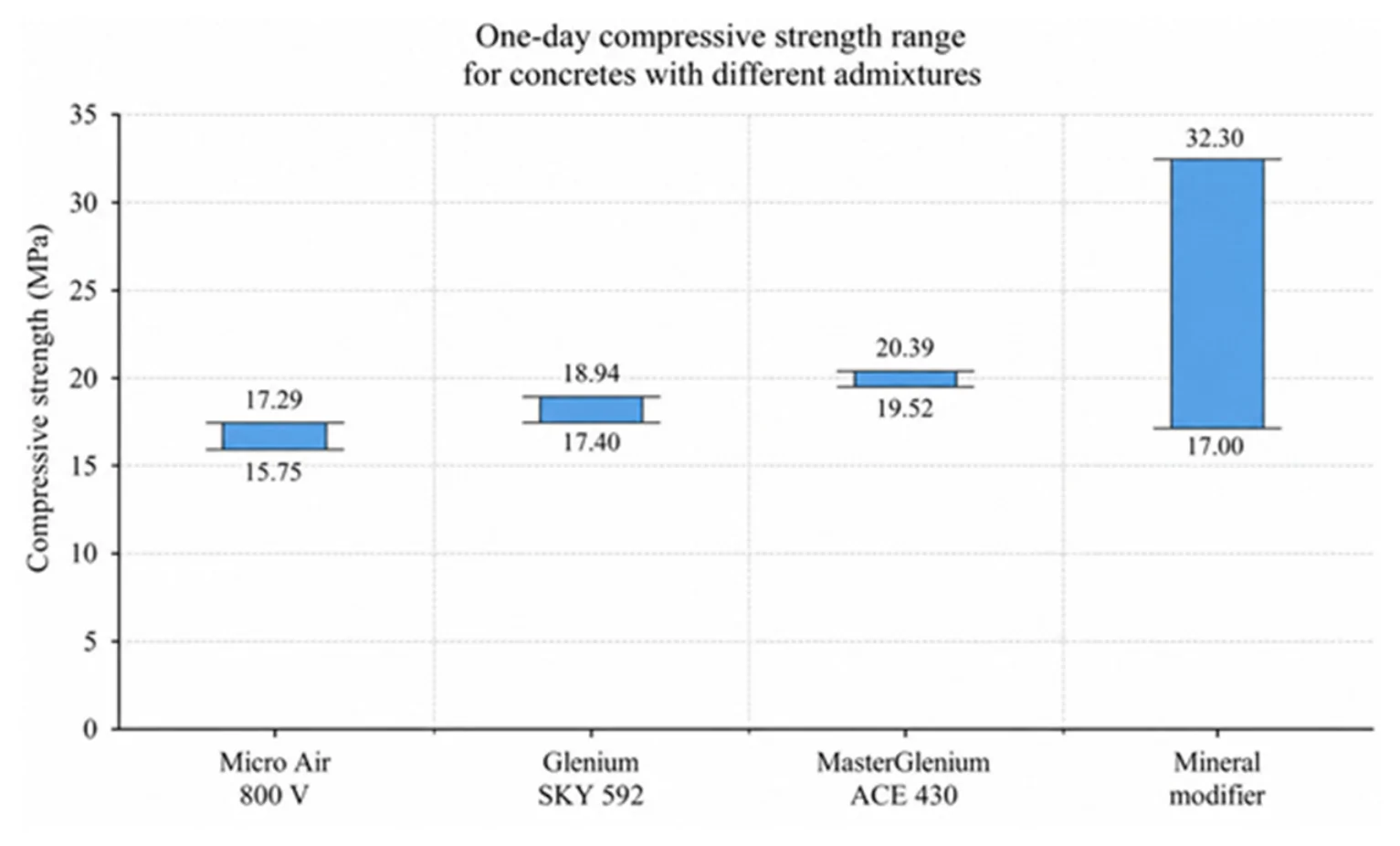
One-day compressive strength range for concretes prepared with different admixture systems.

**Table 1:** Chemical composition of 5–20 mm crushed high-carbon slag aggregate.

Measured Characteristic Unit	Measured Value
Mass fraction of CaO %	not determined
Mass fraction of SiO_2_ %	27.79
Mass fraction of Al_2_O_3_ %	15.85
Mass fraction of MgO %	43.83
Mass fraction of FeO %	0.82
Mass fraction of MnO %	0.099
Mass fraction of SO_3_ %	0.07
Mass fraction of SO_4_ %	0.27
Mass fraction of SO_3_ + SO_4_ %	0.34
Mass fraction of P_2_O_5_ %	not determined

Measurements were performed using an Axios 1 kW wavelength-dispersive X-ray fluorescence spectrometer (PANalytical, Almelo, The Netherlands). The obtained data were processed and interpreted using SuperQ software (Omnian 37). The error of the semi-quantitative analysis was ±20% (relative).

**Table 2:** X-ray fluorescence analysis results for high-carbon slag.

Element Content	Detection	Compound Formula	Quantitative Content	Unit
Na	detected	Na_2_O	0.123	%
Mg	detected	MgO	41.395	%
AI	detected	AI_2_O_3_	20.892	%
Si	detected	SiO_2_	29.906	%
P	detected	P_2_O_5_	0.008	%
S	detected	SO_3_	0.253	%
Ca	detected	CaO	1.119	%
Ti	detected	TiO_2_	1.292	%
V	detected	V_2_O_5_	4.268	%
Cr	detected	Cr_2_O_3_	0.178	%
Mn	detected	MnO	0.546	%
Fe	detected	Fe_2_O_3_	0.008	%
Sr	detected	SrO	0.012	%

**Table 3:** Chemical composition of “carbonate” shungite shale.

**Component**	**C**	**SiO_2_**		**CaO**		**Fe_2_O_3_**		**Al_2_O_3_**		**TiO_2_**		**Na_2_O**
Content. %	12	38		31		2.69		4.55		0.50		1.20
**Component**	**K_2_O**		**Mn**		**Zn**		**Cu**		**Ba**		**LOI**	
Content. %	0.40		0.09		0.01		0.01		0.05		9.51	

**Table 4:** Fineness of the ground modifier as a function of grinding time and grinding body type.

Sieve No.	Nominal Clear Mesh Size. mm	Sieve Residue After Grinding %	Sieve Residue After Grinding %	Sieve Residue After Grinding %	Sieve Residue After Grinding %	Sieve Residue After Grinding %	Sieve Residue After Grinding %
Sieve No.	Nominal clear mesh size. mm	30 min	30 min	45 min	45 min	60 min	60 min
Sieve No.	Nominal clear mesh size. mm	balls	cylpebs	balls	cylpebs	balls	cylpebs
09	0.900	-	-	-	-	-	-
008	0.080	9.4	7.5	9.1	7.3	9.0	7.1
>008	0.080	90.6	92.5	90.9	92.7	91.0	92.9

**Table 5:** Pozzolanic activity of the mineral modifying additive based on tailings, silica fume, and shungite waste by CaO absorption after 28 days.

Mineral Additive Composition %	Mineral Additive Composition %	Mineral Additive Composition %	CaO Absorption After 28 Days %
processing tailings	silica fume	shungite waste	CaO absorption after 28 days. %
99.8	0	0.2	7.9
89.6	10	0.4	13.0
79.4	20	0.6	25.9
69.2	30	0.8	57.4
59	40	1.0	59.2
48.8	50	1.2	63.5
38.6	60	1.4	68.2
28.4	70	1.6	74.3
18.2	80	1.8	80.9
8	90	2.0	84.6
0	100	-	94.0

**Table 8:** Capillary water absorption results for the developed concrete compositions.

Capillary Absorption. %	Mixture No. 1	Mixture No. 2	Mixture No. 3	Mixture No. 4	Mixture No. 5	Mixture No. 6
After 60 min	0.72	0.92	0.53	0.54	1.03	1.12
After 12 h	3.35	3.16	3.07	2.98	3.79	4.10
After 24 h	4.82	4.30	4.13	4.26	5.03	5.17
After 48 h	5.32	5.08	4.30	4.42	5.66	5.83

**Table 9:** Freeze–thaw resistance results for the developed concrete compositions.

Freeze–Thaw Resistance	Mixture No. 1	Mixture No. 2	Mixture No. 3	Mixture No. 4	Mixture No. 5	Mixture No. 6
Freeze–thaw cycles; compressive strength criterion; mass loss. %	75/56.92 > 54.43/1.6	75/55.89 > 52.39/1.8	110/60.56 > 57.28/1.9	110/58.56 > 56.84/1.8	75/53.95 > 51.72/1.5	75/52.40 > 50.03/1.7
Freeze–thaw grade	F300	F300	F400	F400	F300	F300

## Data Availability

The original contributions presented in this study are included in the article. Further inquiries can be directed to the corresponding authors.
